# Integrated Network Toxicology and Metabolomics Reveal the Reproductive Toxicity Mechanisms of Alkaloid-Enriched Fractions in Tripterygium Glycosides Tablets

**DOI:** 10.3390/toxins18040175

**Published:** 2026-04-04

**Authors:** Caiyao Han, Hong Yuan, Sixian Chen, Zhuohua Huang, He Gong, Lin Lv, Xinpeng Zhou, Jiang Ma, Xin He

**Affiliations:** 1 School of Chinese Materia Medica, Guangdong Pharmaceutical University, Guangzhou 510006, China; hancyxw@163.com (C.H.); yuanhong20211012@163.com (H.Y.); chensixian2026@163.com (S.C.); hzh815361670@163.com (Z.H.); gonghehg@163.com (H.G.); gonggong9975@163.com (L.L.); sym_ple17151@163.com (X.Z.); 2 Key Specialty of Clinical Pharmacy, The First Affiliated Hospital of Guangdong Pharmaceutical University, Guangzhou 510080, China; 3 Guangdong Provincial Key Laboratory for Research and Evaluation of Pharmaceutical Preparations, Guangdong Pharmaceutical University, Guangzhou 510006, China

**Keywords:** tripterygium glycoside tablets, alkaloid, reproductive toxicity, network toxicology, metabolomics

## Abstract

Tripterygium glycoside tablets (TGT), a representative formulation derived from *Tripterygium wilfordii* Hook F, have limited clinical application due to adverse reproductive toxicity. In previous studies investigating the effects of TGT on chronic kidney disease (CKD), it was found that both TGT and its alkaloid-enriched fraction (AEF) induced testicular atrophy, suggesting that AEF may be the material basis for the reproductive toxicity of TGT. Therefore, the reproductive toxicity of AEF was investigated in depth. This study established a CKD rat model to investigate the toxic effects of TGT, AEF, and the non-alkaloid-enriched fraction (NAEF) on the reproductive system during CKD treatment. Network toxicology and metabolomics were combined to elucidate the underlying mechanisms of AEF-induced reproductive toxicity. The results showed that both TGT and AEF significantly reduced testicular index and sperm concentration, causing seminiferous tubule atrophy and disrupting the levels of testosterone (T), follicle-stimulating hormone (FSH), and luteinizing hormone (LH). Furthermore, TGT, AEF, and NAEF all significantly inhibited the proliferation of GC-1 cells. Network toxicology indicated that AEF modulates targets such as SRC, AKT, and HSP90AA1, thereby influencing pathways including the PI3K-AKT signaling pathway and pathways in cancer. Metabolomics obtained 89 differential metabolites of AEF, which were enriched in glycerophospholipid, linoleic acid, and arachidonic acid metabolism, a finding consistent with the constructed “metabolite–enzyme–reaction–gene” network. In summary, AEF exerts reproductive toxicity primarily by disrupting hypothalamic–pituitary–testicular axis homeostasis and perturbing glycerophospholipid, linoleic acid, and arachidonic acid metabolism.

## 1. Introduction

*Tripterygium wilfordii* (*T*. *wilfordii*), a traditional Chinese herbal medicine, has been widely used for the treatment of autoimmune and inflammatory diseases, including rheumatoid arthritis and systemic lupus erythematosus. However, *T. wilfordii* is also among the herbal medicines most frequently associated with adverse drug reactions, particularly reproductive and hepatorenal toxicities, which have greatly limited its clinical application [1,2,3]. Tripterygium glycosides (TG), the total glycoside fraction extracted from the peeled roots of *T. wilfordii*, contain bioactive diterpenes, triterpenes, and alkaloids that exhibit immunoregulatory, anti-inflammatory, and antioxidative activities, and have been extensively applied in traditional Chinese medicine practice [3,4]. Tripterygium glycoside tablets (TGT), a representative preparation of *T. wilfordii*, are used as first–line agents for autoimmune diseases such as systemic lupus erythematosus and nephrotic syndrome. Nevertheless, their reproductive toxicity has raised considerable concern [5,6]. Epidemiological data report an overall incidence of reproductive toxicity of 17.9% among TGT users, with rates of 15.7% in adults and 24.4% in children, which significantly limits its therapeutic use [7]. Sperm impairment is recognized as the major manifestation of TGT-induced toxicity, characterized by reduced motility, oligospermia, or azoospermia, potentially leading to infertility [8,9,10]. Mechanistically, the reproductive toxicity of TG has been linked to decreased sperm quality and serum sex hormones, testicular injury, impaired spermatogenesis, disturbed energy metabolism, dysregulation of the PI3K-Akt and AMPK/mTOR pathways, disruption of the hypothalamic–pituitary–testicular (HPT) axis, and ferroptosis induction [3,11].

Network toxicology, an emerging discipline integrating network pharmacology and systems biology, combines bioinformatics and big data analytics with genomics, proteomics, and metabolomics to explore the toxicological pathways of compounds and elucidate the molecular mechanisms underlying disease-related toxicity [12,13]. This network-based approach elucidates how diverse molecules interact within biological systems and how these interactions lead to toxicity. By integrating multiple data sources, the relationships among compounds, toxic effects, and molecular targets can be systematically mapped, providing a systems-level understanding of complex toxicological mechanisms and facilitating the prediction of potential targets and disease–drug interactions [13,14].

Metabolomics, a powerful and rapidly developing analytical technique, enables comprehensive profiling of low-molecular-weight metabolites (<1 kDa) in biological systems [15]. This approach provides a noninvasive, sensitive, and efficient strategy for evaluating the toxicity of traditional Chinese medicines by identifying endogenous biomarkers associated with toxic responses. The fundamental principle is that toxins disrupt cellular structure and function, leading to detectable perturbations in endogenous metabolic pathways. By analyzing the correlations between physiological states and metabolic alterations, metabolomics can be used to evaluate herbal toxicity, discover related biomarkers, and elucidate toxic mechanisms. This contributes to rational clinical use and the reduction of adverse reactions. In toxicological studies of traditional Chinese medicines, metabolomics plays an increasingly vital role in rapidly assessing toxicity and clarifying its mechanisms by linking drug-induced physiological responses to metabolic phenotypes [15,16,17,18].

Current studies on the reproductive toxicity of TGT have mainly focused on non-alkaloid components such as triptolide [19,20] and tripdiolide [21]. In our previous work, the alkaloid-enriched fraction (AEF) and non-alkaloid-enriched fraction (NAEF) of TGT were enriched in a ratio of 2.8:1. During investigations of the renoprotective effects of TGT, AEF, and NAEF in chronic kidney disease (CKD), we observed that TGT and AEF induced testicular lesions in rats, suggesting that, in addition to NAEF, AEF might also possess reproductive toxicity. Based on these findings, we further explored the reproductive toxicity of AEF in depth. Using metabolomics techniques, we precisely characterized the specific mechanisms of AEF-induced reproductive toxicity after excluding the influence of CKD. Combined with network toxicology analysis, we identified the key molecular targets most likely to mediate these toxic effects and constructed a comprehensive mechanistic framework linking “drug–target” interactions to “metabolic disturbance” and the resulting “toxic phenotype”. This study aims to provide novel insights into the prevention and mitigation of TGT-induced reproductive toxicity, thereby enhancing clinical safety while preserving therapeutic efficacy.

## 2. Results

### 2.1. Effects of the AEF of TGT on Reproductive Toxicity in Rats

**Figure 1:**
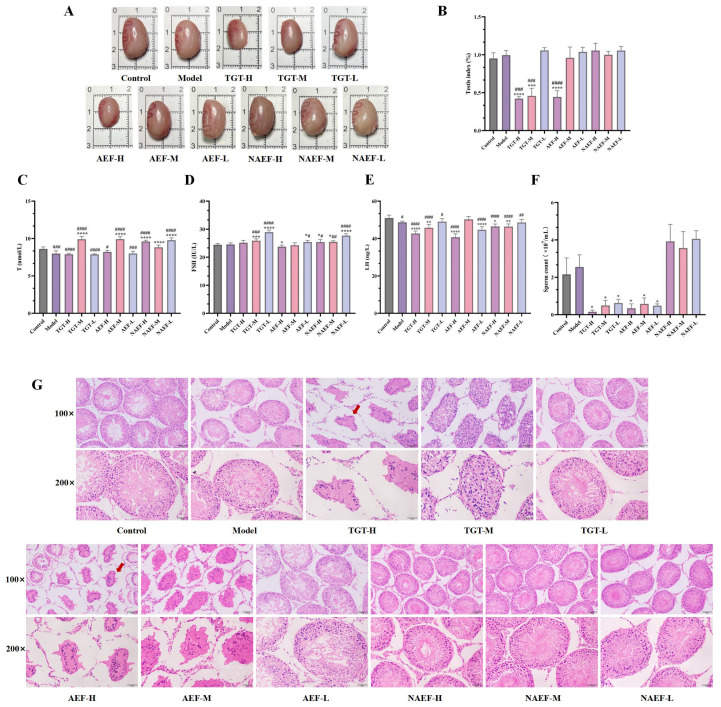
Effects of the AEF of TGT on reproductive toxicity in rats. Note: (**A**) Morphology of rat testes; (**B**) Testicular index (*n* = 6); (**C**) Plasma T levels in each group (*n* = 6); (**D**) Plasma FSH levels in each group (*n* = 6); (**E**) Plasma LH levels in each group (*n* = 6); (**F**) Sperm concentration (*n* = 6); (**G**) Histopathological observations of testicular tissue (Red arrows: seminiferous tubule atrophy and deformation). Compared with the control, ^#^ *p* < 0.05, ^##^ *p* < 0.01, ^###^ *p* < 0.001, ^####^ *p* < 0.0001; compared with the model, * *p* < 0.05, ** *p* < 0.01, *** *p* < 0.001, **** *p* < 0.0001.

As shown in Figure 1A, the morphology of the testicular tissue varied among the groups. The testes in the control group appeared smooth and intact, whereas those in the TGT-H, TGT-M, AEF-H, and AEF-M groups showed evident shrinkage, and the surface of the tunica albuginea was no longer smooth. No apparent pathological changes were observed in the other groups. Analysis of the testicular index (Figure 1B) revealed no significant difference between the control and model groups (*p* > 0.05). However, compared with both the control and model groups, the testicular index was significantly decreased in the TGT-H, TGT-M, and AEF-H groups (*p* < 0.001 or *p* < 0.0001).

The ELISA results for plasma testosterone (T) levels (Figure 1C) indicated that, compared with the control group, T levels were significantly decreased in the model, TGT-H, TGT-L, AEF-H, and AEF-L groups (*p* < 0.05, *p* < 0.001 or *p* < 0.0001), while TGT-M, AEF-M, NAEF-H, and NAEF-L groups showed significantly increased levels (*p* < 0.0001). Compared with the model group, T levels were significantly elevated in the TGT-M, AEF-M, and all NAEF groups (*p* < 0.0001). The follicle-stimulating hormone (FSH) levels (Figure 1D) showed no significant difference between the control and model groups (*p* > 0.05). However, FSH levels were significantly increased in the TGT-M, TGT-L, AEF-L, and all NAEF groups compared with both control and model groups (*p* < 0.05, *p* < 0.01, *p* < 0.001 or *p* < 0.0001), while AEF-H exhibited a significant decrease (*p* < 0.05). For luteinizing hormone (LH) (Figure 1E), the model, TGT, and NAEF groups at all doses, as well as AEF-H and AEF-L, showed significantly reduced levels compared with the control group (*p* < 0.05, *p* < 0.01 or *p* < 0.0001). Relative to the model group, LH levels were also significantly decreased in the TGT-H, TGT-M, AEF-H, AEF-L, NAEF-H, and NAEF-M groups (*p* < 0.05, *p* < 0.01 or *p* < 0.0001).

Sperm concentration results (Figure 1F) revealed no significant difference between the model and control groups (*p* > 0.05). However, compared with the model group, sperm concentrations were significantly reduced in all TGT and AEF dose groups (*p* < 0.05). Histopathological examination of the testes (Figure 1G) showed that the model group had seminiferous tubules similar to those in the control group, with orderly cell arrangement and visible spermatozoa in the lumen. In contrast, TGT-H, TGT-M, AEF-H, and AEF-M groups exhibited severe seminiferous tubule atrophy and deformation (indicated by red arrows), irregular shapes, widened interstitial spaces, disorganized spermatogenic cells, and absence of mature spermatozoa. The NAEF groups showed regular seminiferous tubule morphology with orderly cell arrangement and visible spermatozoa, resembling those of the control group.

### 2.2. The Effect of TGT, AEF and NAEF on GC-1

**Figure 2:**
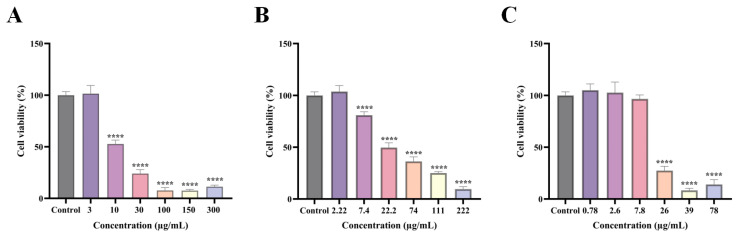
Effects of TGT, AEF, and NAEF on cell viability. Note: (**A**) Different concentrations of TGT; (**B**) Different concentrations of AEF; (**C**) Different concentrations of NAEF. Compared with the control, **** *p* < 0.0001.

To further confirm the reproductive toxicity of AEF, cell viability was assessed using the Cell Counting Kit-8 (CCK-8) assay. Based on the previously determined ratio of AEF to NAEF components in TGT, GC-1 cells were treated with corresponding gradient concentrations. As shown in Figure 2, TGT exhibited cytotoxic effects on GC-1 cells beginning at 10 μg/mL, with increasing toxicity observed up to 150 μg/mL in a concentration-dependent manner. The AEF showed toxicity starting at 7.4 μg/mL, which also intensified with increasing concentration. In contrast, the NAEF exhibited detectable cytotoxicity only at concentrations ≥26 μg/mL, and its dose–response relationship requires further investigation. Collectively, these results indicate that the AEF induces testicular damage and impairs reproductive development in male rats, demonstrating clear reproductive toxicity that may be dose-dependent.

### 2.3. Network Toxicology Analysis Results

#### 2.3.1. Prediction of Alkaloid Components and Reproductive Toxicity-Related Targets

**Figure 3:**
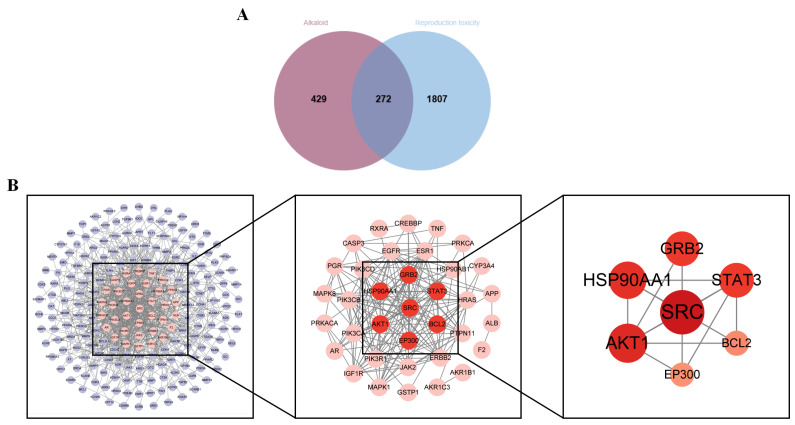
Overlapping Targets and PPI Network Analysis. Note: (**A**) Venn diagram of alkaloid-reproductive toxicity targets; (**B**) Visualization of the PPI network and core targets.

Based on literature reports and previous studies conducted by our group, a total of 33 alkaloid components were identified from TGT. Using multiple online databases, 701 potential targets corresponding to 23 alkaloid components were obtained (Table 1). These were intersected with 2079 reproductive toxicity-related targets, resulting in 272 overlapping targets (Figure 3A).

#### 2.3.2. Protein–Protein Interaction Network Analysis

The 272 intersecting targets were imported into the STRING database to construct a protein–protein interaction (PPI) network, which consisted of 272 nodes and 796 edges, where nodes represent proteins and edges indicate interactions between them. The PPI network was visualized using Cytoscape software (3.10.0), and topological parameters—including Degree, Closeness, and Betweenness—were calculated. The top 20 targets for each metric were intersected, yielding seven core targets; namely, SRC, AKT1, HSP90AA1, GRB2, STAT3, BCL2, and EP300 (Figure 3B).

#### 2.3.3. Gene Ontology and Kyoto Encyclopedia of Genes and Genomes Analyses

**Figure 4:**
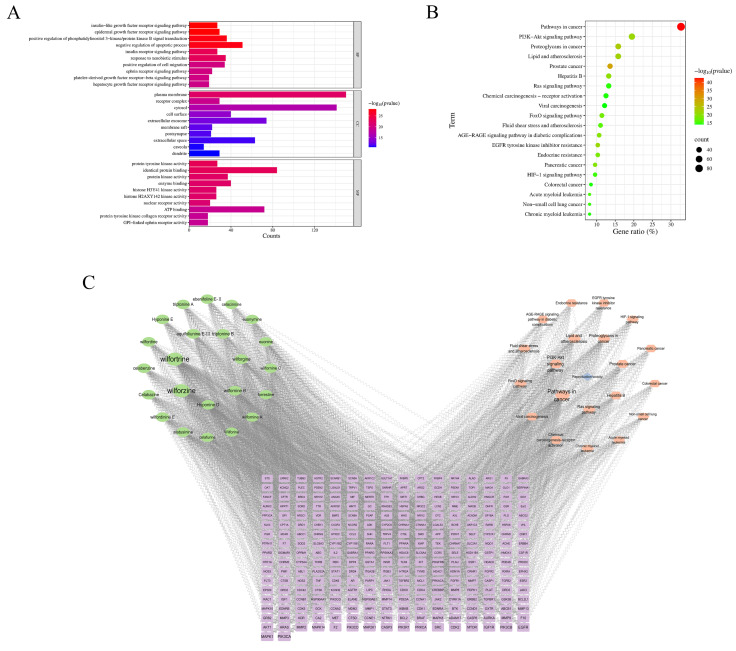
GO and KEGG enrichment analyses and “alkaloid–targets–pathways” network. Note: (**A**) Bar plot of GO enrichment analysis; (**B**) Bubble plot of KEGG pathway enrichment; (**C**) “alkaloid components–targets–pathways” network.

Gene Ontology (GO) analysis was performed to categorize the intersecting targets into biological processes (BP), cellular components (CC), and molecular functions (MF). A total of 1444 GO terms were obtained, among which 1142 terms met the criterion (*p* ≤ 0.05), including 797 BP, 112 CC, and 233 MF terms. The top ten terms from each category were visualized in bar charts (Figure 4A). Kyoto Encyclopedia of Genes and Genomes (KEGG) pathway enrichment analysis identified 183 signaling pathways, of which 172 were significantly enriched (*p* ≤ 0.05). The top 20 pathways, ranked by *p*-value and gene count, were visualized in a bubble plot (Figure 4B). In the plot, darker colors indicate higher confidence, and larger dots represent pathways containing more genes.

#### 2.3.4. Construction of the “Alkaloid Components–Reproductive Toxicity Targets–Pathways” Network

The “alkaloid components–reproductive toxicity targets–pathways” network was constructed using Cytoscape software (3.10.0) (Figure 4C). In the network, purple rectangles represent reproductive toxicity-related targets, green ellipses represent alkaloid components, blue diamonds indicate reproductive toxicity, and pink hexagons represent signaling pathways. The larger the node, the higher the Degree value and the greater its importance in the network. Based on a Degree value ≥ 83, seven major toxic alkaloid components were identified: wilforzine, wilfortrine, aquifoliunine E-III, triptonine B, wilforgine, hyponine D, and wilfornine B.

### 2.4. Untargeted Metabolomics Analysis Results

#### 2.4.1. Principal Component Analysis

**Figure 5:**
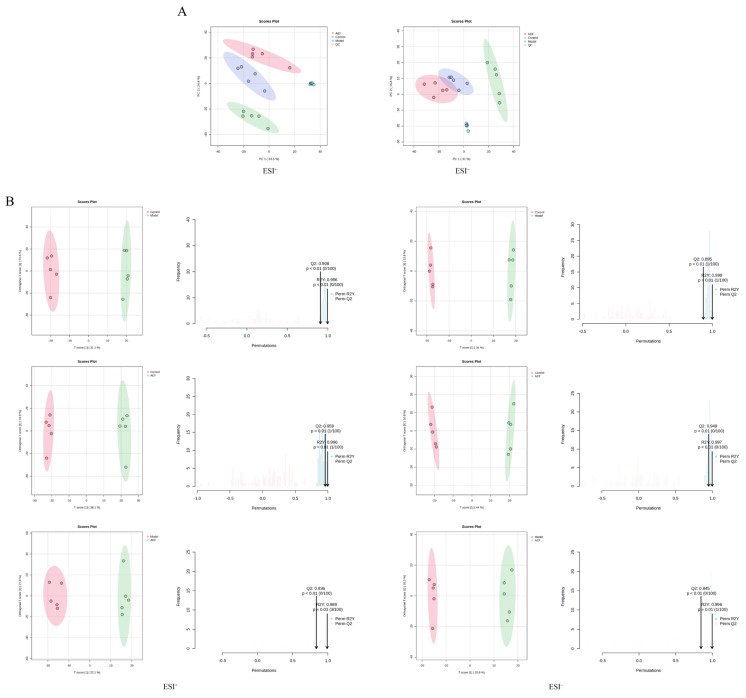
PCA and OPLS−DA analyses of plasma metabolites. Note: (**A**) PCA score plots in positive and negative ion modes; (**B**) OPLS−DA score plots showing group separations and model validation. AEF: Alkaloid-enriched fraction; QC: Quality control.

An unsupervised principal component analysis (PCA) was performed on plasma samples from each group, and the results are shown in Figure 5A. In both positive and negative ion modes, the QC samples were tightly clustered, indicating good system stability and reproducibility. The control and model groups each formed distinct and well-separated clusters, suggesting that CKD induction caused substantial metabolic disturbances. The AEF group formed a third cluster, clearly separated from both the control and model groups, with no evidence of metabolic reversion under the disease background. This finding suggests that the AEF group underwent a new pattern of metabolic regulation in the CKD context. Based on previous findings on reproductive toxicity, we hypothesize that this metabolic pattern reflects a systemic manifestation of AEF-induced toxicity. Therefore, subsequent differential metabolite analyses focused on characterizing this specific metabolic alteration.

#### 2.4.2. Orthogonal Partial Least Squares−Discriminant Analysis

A supervised orthogonal partial least squares−discriminant analysis (OPLS-DA) was further conducted, and the results are shown in Figure 5B. After 100 permutation tests, the following model parameters were obtained.

In positive ion mode: control vs. model group (*R*^2^Y = 0.996, *Q*^2^ = 0.908); control vs. AEF group (*R*^2^Y = 0.996, *Q*^2^ = 0.959); model vs. AEF group (*R*^2^Y = 0.989, *Q*^2^ = 0.836). In negative ion mode: control vs. model group (*R*^2^Y = 0.999, *Q*^2^ = 0.895); control vs. AEF group (*R*^2^Y = 0.997, *Q*^2^ = 0.949); model vs. AEF group (*R*^2^Y = 0.996, *Q*^2^ = 0.845). These results demonstrate that the models were highly stable and exhibited strong predictive capability.

#### 2.4.3. Differential Metabolite Analysis

**Figure 6:**
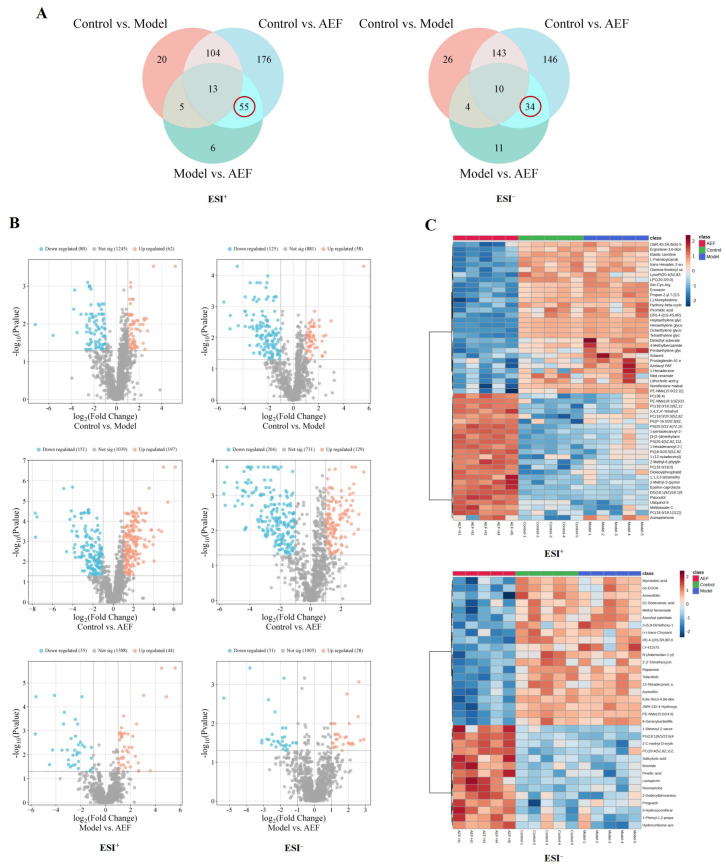
Differential metabolite analysis. Note: (**A**) Venn diagram of differential metabolites (red circles: reproductive toxicity−related metabolites); (**B**) Volcano plots showing metabolite changes between groups; (**C**) Heatmaps of reproductive toxicity−related metabolites.

To eliminate the metabolic background of CKD and specifically identify AEF-induced toxic signatures, a Venn diagram analysis was performed. Differential metabolites obtained from comparisons of control vs. model, control vs. AEF, and model vs. AEF groups were visualized and intersected. Metabolites that showed significant changes in both “control vs. AEF” and “model vs. AEF” comparisons but no significant change in “control vs. model” were defined as CKD background-excluded specific reproductive toxicity metabolites and were used for subsequent pathway enrichment analysis (Figure 6A). Based on OPLS-DA, differential metabolites were screened using the criteria VIP > 1, *p* < 0.05, and fold change (FC) ≥ 2 or ≤ 0.5. In positive ion mode, 142 differential metabolites were identified between the control and model groups, 348 between the control and AEF groups, and 79 between the model and AEF groups, resulting in 55 reproductive toxicity-related metabolites (volcano plot shown in Figure 6B, heatmap in Figure 6C, details in Table 2). In negative ion mode, 183 differential metabolites were identified between the control and model groups, 333 between the control and AEF groups, and 59 between the model and AEF groups, yielding 34 reproductive toxicity-related metabolites (Figure 6B,C, and Table 3).

#### 2.4.4. Metabolic Pathway Analysis

**Figure 7:**
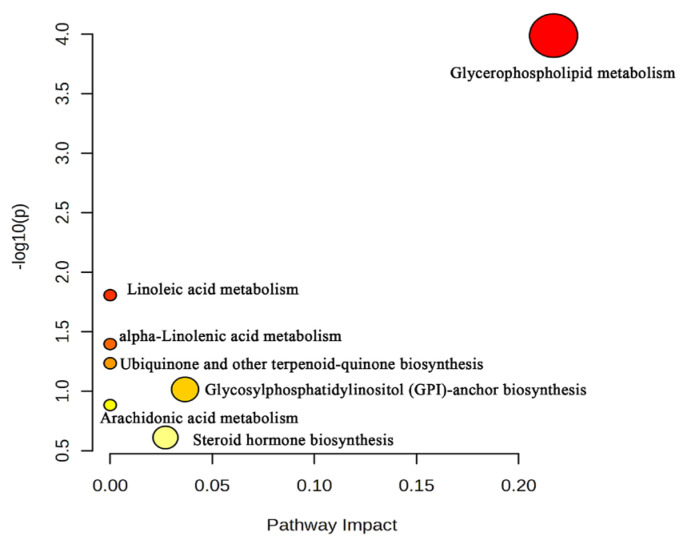
KEGG pathway enrichment plot.

All reproductive toxicity-related metabolites identified in both ionization modes were integrated and analyzed for KEGG pathway enrichment using the MetaboAnalyst (6.0) platform, and the results were visualized as a bubble plot (Figure 7). Seven enriched pathways were identified: glycerophospholipid metabolism, linoleic acid metabolism, alpha-linolenic acid metabolism, ubiquinone and other terpenoid-quinone biosynthesis, glycosylphosphatidylinositol (GPI)-anchor biosynthesis, arachidonic acid metabolism, and steroid hormone biosynthesis.

### 2.5. Integration of Network Toxicology and Metabolomics

**Figure 8:**
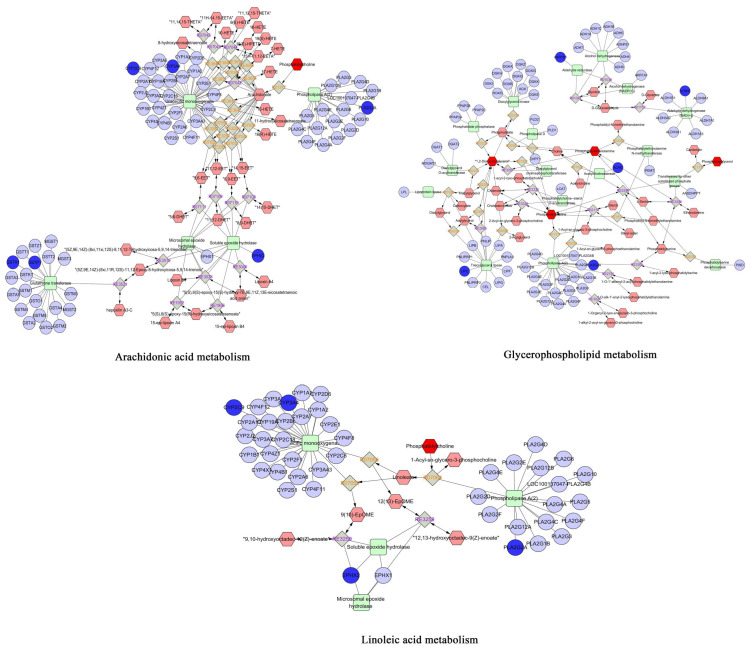
Integrated “metabolite–enzyme–reaction–gene” network based on network toxicology and metabolomics.

The specific reproductive toxicity-related metabolites identified above were further correlated with the intersecting targets from network toxicology analysis. A comprehensive “metabolite–enzyme–reaction–gene” network was constructed using Cytoscape software (3.10.0) (Figure 8). The results revealed that glycerophospholipid metabolism, linoleic acid metabolism, and arachidonic acid metabolism were consistent with the findings of metabolomic pathway enrichment, suggesting that these pathways may play crucial roles in AEF-induced reproductive toxicity of TGT.

## 3. Discussion

TG, the major active components extracted from the traditional Chinese medicinal plant *Tripterygium wilfordii* Hook F, possess traditional pharmacological properties such as dispelling wind and dampness, promoting blood circulation, alleviating pain and swelling, and detoxification. Historically, TG has been used to treat dermatological conditions such as leprosy and eczema [11]. In modern clinical practice, TGT, as a potent non-steroidal immunosuppressive agent, have been widely applied in the treatment of rheumatoid arthritis, nephritis, ankylosing spondylitis, and various autoimmune diseases, exhibiting strong anti-inflammatory and antitumor activities [20]. However, despite its notable therapeutic efficacy, TGT is also associated with serious and wide-ranging toxicities, including multi-organ damage such as hepatotoxicity, nephrotoxicity, and reproductive toxicity (manifested as male infertility and menstrual disorders), as well as leukopenia. These adverse effects have severely restricted its broader clinical application [8,9,22].

Clinical studies have confirmed that TGT reduces sperm count and density, and long-term administration can result in testicular atrophy [8,23]. TGT mainly consists of diterpenoids, triterpenoids, and alkaloids [24]. In our previous study investigating the effects of TGT, AEF, and NAEF on CKD, we observed evident testicular atrophy in the high- and medium-dose TGT groups and the high-dose AEF group. This led us to hypothesize that the AEF also contributes to its reproductive toxicity. Further assessments of the testicular index and sperm concentration revealed significant decreases compared with both the control and model groups, accompanied by histological evidence of testicular injury.

Biochemical assays showed that FSH levels were significantly elevated in the TGT-M/L, AEF-L, and all NAEF dose groups compared with the control group, which may be attributed to damage of Sertoli cells. Under normal physiological conditions, inhibin B secreted by Sertoli cells exerts negative feedback on FSH secretion, and their levels are inversely correlated. When Sertoli cell function is impaired, inhibin B secretion decreases, thus relieving the inhibitory feedback on pituitary FSH release, leading to compensatory elevation of FSH levels [25,26,27]. In addition, TGT-H/L and Aef-H/L groups exhibited decreased T and LH levels compared with the control group. Normally, FSH binds to receptors on Sertoli cell membranes, activating cAMP-dependent phosphorylation and promoting the synthesis of androgen-binding protein (ABP). ABP specifically binds to T to maintain an appropriate microenvironment for spermatogenesis. Damage to Sertoli cells reduces ABP synthesis and cAMP activity, leading to decreased intratesticular T levels [27]. The reduction in LH may be due to drug-induced suppression of hypothalamic gonadotropin-releasing hormone (GnRH) secretion or reduced pituitary responsiveness to GnRH, leading to decreased LH synthesis and consequently lower T levels [28].

Interestingly, TGT-M and all NAEF groups showed significantly elevated T levels but decreased LH levels compared with the control group. This suggests that TGT or its active metabolites may directly stimulate Leydig cells, promoting T synthesis and secretion. Excessive T then exerts negative feedback on the hypothalamic–pituitary axis, inhibiting GnRH release and reducing LH levels [29,30,31]. Multi-omics analysis by Guo et al. [10] also demonstrated that TGT disrupts testicular steroidogenesis and the Fgf signaling pathway associated with the NELL2-lumicrine system, supporting the hypothesis that its active components may directly interfere with the T synthesis pathway. In addition, T levels in the model group were significantly lower than those in the control group, which may be attributed to disruption of the hypothalamic–pituitary–gonadal (HPG) axis under conditions of renal insufficiency. Impaired renal function reduces the clearance of prolactin, leading to elevated prolactin levels that inhibit the pulsatile secretion of GnRH. Furthermore, the activity of 17β-hydroxysteroid dehydrogenase (17β-HSD) in the testes is suppressed during renal insufficiency, resulting in decreased T synthesis [32,33].

In summary, TGT and AEF can induce complex hormonal disturbances. Based on the available evidence, we hypothesize that these effects may result from the disruption of the functional homeostasis of the HPT axis through multiple direct or indirect pathways. To further verify the reproductive toxicity, a CCK-8 assay was conducted using GC-1 cells. The results showed that TGT, AEF, and NAEF significantly inhibited the proliferation of GC-1 cells. Combining the endocrine phenotypes observed in vivo with the cytotoxic effects in vitro, we conclude that the AEF possesses distinct reproductive toxicity.

In this study, the AEF altered the levels of several metabolites, including phosphatidylcholine (PC), lysophosphatidylcholine (LysoPC), and phosphatidylinositol (PI) in both the control and model groups. Integrated analysis combining network toxicology and metabolomics revealed that AEF may induce male reproductive toxicity primarily by regulating the glycerophospholipid metabolism, linoleic acid metabolism, and arachidonic acid metabolism pathways. LysoPC is a lipid molecule derived from glycerophospholipids (GPLs) and is mainly produced by the hydrolysis of PC via phospholipase A2 (PLA2). LysoPC has been recognized as a biomarker for evaluating sperm quality in both humans and animals. When the sperm plasma membrane is damaged, excessive PLA2 activity can lead to abnormal hydrolysis of PC, resulting in LysoPC accumulation and disruption of the physiological timing of the acrosome reaction. In this study, we observed decreased PC levels and elevated LysoPC levels in the AEF group, indicating that AEF disrupted glycerophospholipid metabolism, impaired sperm quality, and consequently affected the acrosome reaction [34,35,36].

Arachidonic acid (AA), a polyunsaturated fatty acid released by PLA2 activation, serves as a precursor for the synthesis of prostaglandins (PGs) and thromboxanes via the cyclooxygenase (COX) pathway. PGs play essential roles in reproductive regulation by stimulating the secretion of prolactin, LH, and FSH, which in turn promote T synthesis in Leydig cells, regulate spermatogenesis, and improve semen quality. Moreover, AA upregulates lactate dehydrogenase (LDH) mRNA expression, enhancing lactate production and energy supply by Sertoli cells to support spermatogenesis [37,38,39]. Based on these findings, we propose that the reproductive toxicity of AEF is closely related to interference with arachidonic acid metabolism. The underlying mechanism may involve excessive activation of PLA2, leading to abnormal hydrolysis of PC and overproduction of AA. On one hand, excessive AA may disrupt hormonal balance within the HPG axis, indirectly impairing spermatogenesis. On the other hand, AA can be metabolized via the COX and lipoxygenase (LOX) pathways to generate inflammatory mediators such as prostaglandin E2 (PGE2) and leukotriene B4 (LTB4), which trigger inflammatory responses, compromise sperm membrane integrity, and reduce sperm motility [40]. Furthermore, overactivation of PLA2 may also cause excessive release of linoleic acid, resulting in increased production of PGs, leukotrienes, and lipid peroxides during metabolism. These inflammatory mediators and oxidative lipid species can induce local inflammation and aggravate oxidative stress, ultimately diminishing sperm motility [41,42].

Through network toxicology analysis, seven alkaloid components with relatively high degree values were identified, including wilforzine, wilfortrine, aquifoliunine E-III, triptonine B, wilforgine, hyponine D, and wilfornine B, suggesting that these compounds may represent key effector molecules responsible for reproductive toxicity. These compounds are structurally well-defined sesquiterpene macrocyclic alkaloids derived from *T. wilfordii*. It has been reported that this class of compounds exhibits immunosuppressive, anti-inflammatory, and antitumor activities, which are functionally relevant to the treatment of immune-related diseases such as CKD. Wilfortrine and wilforgine have been reported to inhibit both specific and nonspecific immune responses, while wilfortrine may exert antitumor effects through regulation of the Bcl-2/Bax signaling pathway [43,44]. These findings suggest that alkaloid components may contribute, at least in part, to the overall pharmacological effects of Tripterygium preparations.

Combined with the findings of the present study, metabolomics analysis revealed significant alterations in lipid-related pathways, while network toxicology analysis indicated that the relevant targets were enriched in inflammation- and cell survival-related signaling pathways, such as the PI3K-Akt pathway. These results suggest that AEF may participate in male reproductive toxicity through the regulation of inflammatory responses and lipid metabolism. Meanwhile, these signaling pathways are also involved in immunoregulation and anti-inflammatory processes, indicating that AEF may exert therapeutic effects, while its associated pathways may also overlap, to some extent, with those involved in toxic responses. Considering the pathological characteristics of CKD, which include metabolic disturbances and a pro-inflammatory microenvironment, these conditions may further influence the responsiveness of related signaling pathways, thereby potentially amplifying the toxic effects of AEF.

**Figure 9:**
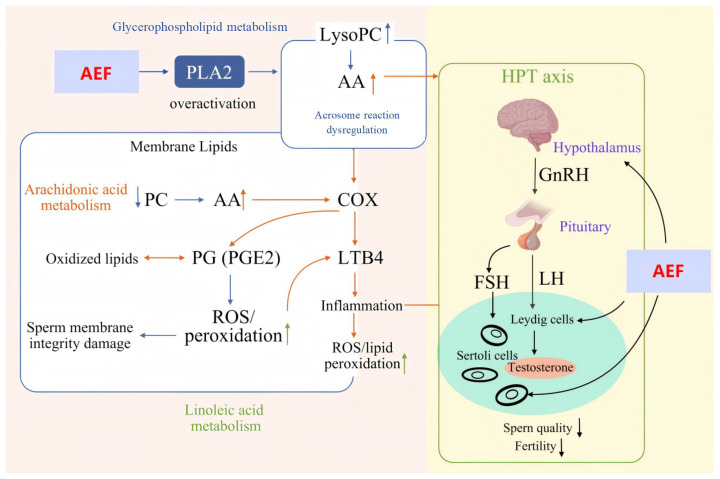
Schematic of the mechanism underlying the reproductive toxicity of AEF in TGT.

Therefore, we propose that AEF primarily induces reproductive toxicity by disrupting the functional homeostasis of the HPT axis and interfering with multiple key metabolic pathways, including glycerophospholipid metabolism, linoleic acid metabolism, and arachidonic acid metabolism. These alterations may subsequently trigger the combined effects of inflammatory responses and oxidative stress, ultimately leading to reproductive toxicity (Figure 9). In addition, it should be noted that TGT is administered as a whole preparation in clinical practice. Based on the enrichment and systematic analysis of AEF in the present study, the findings suggest that the balance between therapeutic effects and toxicity of AEF should be carefully considered during the clinical use of TGT for CKD and other autoimmune diseases. Targeted detoxification strategies may therefore be further explored to reduce adverse effects while maintaining efficacy, thereby providing a reference for optimizing the clinical application of TGT.

## 4. Materials and Methods

### 4.1. Experimental Drugs and Reagents

TGT (batch no. 2305108B) was purchased from Zhejiang Deende Pharmaceutical Co., Ltd. (Zhejiang, China). The AEF and NAEF were prepared in-laboratory. Adenine (purity ≥98%, batch no. C12387667) was purchased from Shanghai Macklin Biochemical Co., Ltd. (Shanghai, China). Sodium carboxymethyl cellulose (batch no. Y24S11X125635) was purchased from Shanghai Yuanye Bio-Technology Co., Ltd. (Shanghai, China). Absolute ethanol (batch no. 100092683) and xylene (batch no. 10023418) were purchased from Sinopharm Chemical Reagent Co., Ltd. (Shanghai, China). Hematoxylin and eosin staining kits (HE, batch no. G1003) and Phosphate-buffered saline (PBS, batch no. GA24080020644) were purchased by Servicebio (Wuhan, China). Rat LH (batch no. MM-0624R1), T (batch no. MM-0577R1), and FSH (batch no. MM-70867R1) ELISA kits were purchased from Guangzhou YANSAI Biological Products Co., Ltd. (Guangzhou, China). Cell Counting Kit-8 (CCK-8, batch no. MA0218-Nov-19J) was purchased from Dalian Meilun Biotechnology Co., Ltd. (Dalian, China). Penicillin–streptomycin solution (batch no. 234137), DMEM medium (batch no. 6124404), and fetal bovine serum (batch no. 012C-0907A) were purchased from Gibco (Thermo Fisher Scientific, Waltham, MA, USA). LC-MS-grade methanol (batch no. I1101035028) and acetonitrile (batch no. I1138629111) were purchased from Merck (Darmstadt, Germany), and LC-MS-grade formic acid (batch no. RH470408) was purchased by Shanghai ANPEL Scientific Instrument Co., Ltd. (Shanghai, China).

### 4.2. Experimental Instruments

Multiskan full-wavelength microplate reader (Thermo Fisher Scientific, Waltham, MA, USA); L420-A low-speed benchtop centrifuge (Xiangyi Instrument Development Co., Ltd., Changsha, China); Donatello tissue dehydrator (Danjier Electronics Co., Ltd., Jinan, China); JB-P5 embedding machine (JunJie Electronics Co., Ltd., Wuhan, China); RM2016 microtome (Leica Instruments Co., Ltd., Shanghai, China); GFL-230 oven (Laiborei Instrument Equipment Co., Ltd., Tianjin, China); LC-30A ultra-high-performance liquid chromatography system (Shimadzu, Kyoto, Japan); Triple TOF 6600+ mass spectrometer (SCIEX Analytical Instruments Trading Co., Ltd., Shanghai, China); 5424R centrifuge (Eppendorf AG, Hamburg, Germany); MU-G02-0448 thermostatic metal mixer (MIULAB Instruments Co., Ltd., Hangzhou, China); VORTEX-5 vortex mixer (Qilinbeier Instrument Manufacturing Co., Ltd., Haimen, China); ACQUITY Premier HSS T3 column (100 mm × 2.1 mm, 1.8 μm; Waters Corporation, Milford, MA, USA).

### 4.3. Experimental Cells

The mouse spermatogonia cell line GC-1 was obtained from Guangzhou Kefan Biotechnology Co., Ltd. (Guangzhou, China). Cells were cultured in DMEM supplemented with 10% FBS under 95% humidity, 5% CO_2_, and 37 °C.

### 4.4. Experimental Animals and Treatment

Male specific-pathogen-free (SPF) Sprague Dawley rats (180–200 g) were purchased from Guangzhou Ruige Biotechnology Co., Ltd. (Guangzhou, China; License No. SCXK [Yue] 2021-0059). The rats were housed under a 12 h light/dark cycle at 20–24 °C and 40–60% humidity with free access to food and water. All animal procedures were approved by the Animal Ethics Committee of Guangdong Pharmaceutical University (Approval No. gdpulacspf 2022439).

Rats were randomly divided into 11 groups (*n* = 8): control, model, TGT high-, medium-, and low-dose groups (TGT-H, TGT-M, TGT-L), AEF high-, medium-, and low-dose groups (AEF-H, AEF-M, AEF-L), and NAEF high-, medium-, and low-dose groups (NAEF-H, NAEF-M, NAEF-L). Except for the control group, all rats received 200 mg/kg adenine by gavage for 3 weeks to induce CKD.

After modeling, the control and model groups received 0.5% CMC-Na, while the treatment groups received TGT extract (90, 30, and 10 mg/kg), AEF (65.7, 21.9, and 7.3 mg/kg), or NAEF (24.3, 8.1, and 2.7 mg/kg) by gavage for 4 weeks. At the end of treatment, rats were anesthetized with isoflurane, and blood samples were collected from the abdominal aorta into anticoagulant tubes. Samples were allowed to stand for 1 h at room temperature, centrifuged at 3000 r/min for 10 min, and the plasma supernatant was stored at −80 °C. Testes were excised, photographed, and weighed to calculate the testicular index (testis weight/body weight).

### 4.5. T, FSH, and LH Content Detection

Plasma levels of T, FSH, and LH were determined using commercial ELISA kits according to the manufacturer’s instructions.

### 4.6. Sperm Concentration Analysis

Epididymal tissues were excised and minced in 2 mL of saline, incubated at 37 °C for 30 min to liquefy, and gently agitated to release sperm. The suspension was diluted tenfold and examined under an optical microscope. Ten random fields were selected for sperm counting, and the sperm concentration was calculated accordingly.

### 4.7. Histopathological Analysis of Testicular Tissue

Testes were fixed in 4% paraformaldehyde, dehydrated in graded ethanol, embedded in paraffin, and sectioned. Sections were stained with hematoxylin and eosin and examined under a light microscope for morphological changes.

### 4.8. Cytotoxicity Assay

GC-1 cells were thawed rapidly in a 37 °C water bath and centrifuged at 1000 rpm for 3 min at 4 °C. The cells were resuspended in complete medium and cultured under 5% CO_2_ at 37 °C. Subculturing was performed at 1:3 when cell density reached 80–90%.

Cell viability was assessed using a CCK-8. Cells in the logarithmic growth phase were counted and seeded into 96-well plates at a density of 5 × 10^3^ cells per well, followed by incubation for 24 h. The medium was then replaced with 100 μL of drug-containing medium at the indicated concentrations: TGT (3, 10, 30, 100, 150, and 300 μg/mL), AEF (2.22, 7.4, 22.2, 74, 111, and 222 μg/mL), and AEF (0.78, 2.6, 7.8, 26, 39, and 78 μg/mL). After 24 h of treatment, 100 μL of fresh medium containing 10% CCK-8 reagent was replaced in each well, and the cells were incubated for an additional 30 min. Absorbance was measured at 450 nm using a microplate reader, and cell viability was calculated using the following equation: Cell viability (%) = (OD_treated_ − OD_blank_)/(OD_control_ − OD_blank_) × 100%.

### 4.9. Network Toxicology Analysis

#### 4.9.1. Target Prediction of Alkaloid Components

Alkaloid components of TGT were identified through a literature review [24,45,46] and prior laboratory identification. The SMILES structures of these compounds were retrieved from the PubChem database and uploaded to the SwissTargetPrediction platform to predict potential target genes. For compounds without available data in PubChem, their mol2 structures were obtained from the TCMSP database and submitted to the PharmMapper server to predict target proteins and acquire their UniProt IDs. All target proteins were subsequently standardized and validated through the UniProt database.

#### 4.9.2. Screening of Reproductive Toxicity-Related and Intersecting Targets

The keywords “Male reproductive toxicity” and “Testicular toxicity” [43] were used to retrieve related targets from the GeneCards database, with relative scores ≥ twice the median value. In addition, “Testicular Diseases” was used as a keyword to search the Comparative Toxicogenomics Database (CTD) to collect disease-associated targets. A Venn diagram of alkaloid component targets and reproductive toxicity-related gene targets was constructed to identify the intersecting targets.

#### 4.9.3. Construction of PPI Network and Identification of Core Targets

Common targets were imported into the STRING database to construct a PPI network. The network was visualized in Cytoscape (3.10.0), and topological parameters (Degree, Closeness, Betweenness) were calculated. The top 20 nodes for each parameter were intersected to determine core targets.

#### 4.9.4. GO and KEGG Pathway Enrichment Analyses

Common targets were uploaded to the DAVID database (species: *Homo sapiens*) for GO functional and KEGG pathway enrichment analyses. The most significant terms and pathways were ranked by *p*-value and visualized using the Bioinformatics online platform.

#### 4.9.5. Construction of “Alkaloid Components–Reproductive Toxicity Targets–Pathways” Network

The “alkaloid components–reproductive toxicity targets–pathways” network was established in Cytoscape (3.10.0), and major toxic components were screened based on Degree ≥ 83.

### 4.10. Untargeted Metabolomics Analysis

#### 4.10.1. Plasma Sample Preparation

Based on previous research results, AEF-H exhibited the strongest toxicity; therefore, control, model, and AEF-H groups were selected for metabolomic analysis. Plasma samples (50 µL) were mixed with 300 µL of 20% acetonitrile–methanol solution, vortexed, and centrifuged at 12,000 r/min for 3 min. The supernatant was transferred for LC-MS analysis. Equal aliquots of each sample were pooled to create a quality control (QC) sample for evaluating system stability.

#### 4.10.2. Analytical Conditions

Chromatographic conditions: Metabolites were detected using an LC-30A ultra-high-performance liquid chromatography system coupled with a Water ACQUITY Premier HSS T3 column (100 mm × 2.1 mm, 1.8 µm). The flow rate was 0.4 mL/min, the injection volume was 4 µL, and the column temperature was 40 °C. Mobile phase: 0.1% formic acid in water (A)—0.1% formic acid in acetonitrile (B). Gradient elution: 0–2 min, 95–80% A; 2–5 min, 89–40% A; 5–6 min: 40–1% A; 6–7.5 min: 1% A; 7.5–7.6 min: 1–95% A; 7.6–10 min: 95% A.

Mass spectrometry was performed using a Triple TOF 6600+ instrument equipped with an electrospray ionization (ESI) source in both positive and negative ion modes. MS^1^ scan range: 50–1000 Da (0.2 s); MS^2^ scan range: 25–1000 Da (0.04 s). Parameters: ion spray voltage +5 kV/−4 kV, source temperature 550 °C/450 °C, declustering potential ±60 V, collision energy ±30 V, spray gas 50 psi, auxiliary heater 60 psi, curtain gas 35 psi.

#### 4.10.3. Data Processing

Raw data were converted to mzXML format using ProteoWizard (3.0) and processed in XCMS for peak extraction, alignment, and retention time correction. Peaks with missing rates > 50% were removed. Peak intensities were normalized by SVR, and metabolites were annotated through database matching. Multivariate statistical analyses and differential metabolite screening were performed using the MetaboAnalyst platform. Differential metabolites excluding CKD background interference were subjected to pathway enrichment analysis.

### 4.11. Statistical Analysis

All statistical analyses were performed using SPSS 26.0. Data are expressed as mean ± standard deviation (SD). One-way analysis of variance (ANOVA) was used for multiple comparisons. Homogeneous variances were tested by LSD, and heterogeneous variances were analyzed by Tamhane’s T2 test. A *p*-value < 0.05 was considered statistically significant.

## Figures and Tables

**Table 1:** Alkaloid components identified from TGT.

No.	Components	No.	Components
1	Wilforgine	13	Wilfornine A
2	Wilfordine	14	Forrestine
3	Wilforine	15	Ebenifoline E-II
4	Wilfortrine	16	Celabenzine
5	Celafurine	17	Wilfordinine E
6	Alatusinine	18	Wilfornine C
7	Aquifoliunine E-III	19	Euonymine
8	Celabazine	20	Euonine
9	Triptonine B	21	Wilfornine B
10	Celacinnine	22	Hyponine E
11	Wilforzine	23	Hyponine D
12	Triptonine A		

**Table 2:** Differential metabolites related to reproductive toxicity identified in ESI^+^ mode.

No.	HMDB ID	Metabolite	Formula	Control vs. AEF	Model vs. AEF
1	HMDB0061822	Hexaethylene glycol	C_12_H_26_O_7_	↑	↑
2	HMDB0061835	Heptaethylene glycol	C_14_H_30_O_8_	↑	↑
3	HMDB0007218	DG(18:1(9Z)/18:1(9Z)/0:0)	C_39_H_72_O_5_	↓	↓
4	-	Octaethylene glycol	C_16_H_34_O_9_	↑	↑
5	HMDB0094708	Tetraethylene glycol	C_8_H_18_O_5_	↑	↑
6	-	PI(18:0/20:5(5Z,8Z,11Z,14Z,17Z))	C_47_H_81_O_13_P	↓	↓
7	HMDB0006318	Gamma-linolenyl carnitine	C_25_H_43_NO_4_	↑	↑
8	-	4-Methylbenzamide oxime	C_8_H_10_N_2_O	↑	↑
9	HMDB0012438	PS(20:4(5Z,8Z,11Z,14Z)/20:4(5Z,8Z,11Z,14Z))	C_46_H_74_NO_10_P	↓	↓
10	HMDB0007946	1-pentadecanoyl-2-(11Z,14Z-eicosadienoyl)-glycero-3-phosphocholine	C_43_H_82_NO_8_P	↓	↓
11	HMDB0114041	[3-[2-(dimethylamino)ethoxy-hydroxyphosphoryl]oxy-2-hexadecanoyloxypropyl] (Z)-octadec-9-enoate	C_41_H_80_NO_8_P	↓	↓
12	HMDB0062653	1-hexadecanoyl-2-(9Z,12Z-octadecadienoyl)-sn-glycero-3-phospho-D-myo-inositol	C_43_H_79_O_13_P	↓	↓
13	HMDB0014610	Enoxacin	C_15_H_17_FN_4_O_3_	↑	↑
14	HMDB0240774	L-Palmitoylcarnitine	C_23_H_45_NO_4_	↑	↑
15	-	Dimethyl suberate	C_10_H_18_O_4_	↑	↑
16	HMDB0032004	Ergostane-3,6-dione	C_28_H_46_O_2_	↑	↑
17	-	Ser-Cys-Arg	C_12_H_24_N_6_O_5_S_1_	↑	↑
18	HMDB0006464	Elaidic carnitine	C_25_H_47_NO_4_	↑	↑
19	-	Hydroxy-beta-cyclocitral	C_10_H_16_O_2_	↑	↑
20	-	Propan-2-yl 7-[3,5-dihydroxy-2-(3-hydroxy-4-phenoxybut-1-en-1-yl)cyclopentyl]hept-5-enoate	C_25_H_36_O_6_	↑	↑
21	HMDB0010393	LysoPC (20:3/0:0)	C_28_H_52_NO_7_P	↑	↑
22	HMDB0000698	Lithocholic acid glycine conjugate	C_26_H_43_NO_4_	↑	↑
23	-	3,4,3′,4′-Tetrahydrospirilloxanthin	C_42_H_64_O_2_	↓	↓
24	HMDB0006317	trans-Hexadec-2-enoyl carnitine	C_23_H_43_NO_4_	↑	↑
25	HMDB0033910	Acetophenone	C_8_H_8_O	↓	↓
26	-	(2R)-4-[(1S,4S,6R)-1,4-Dihydroxy-2,2,6-trimethylcyclohexyl]-3-buten-2-ol	C_13_H_24_O_3_	↑	↑
27	HMDB0008039	PC(18:0/18:2(9Z,12Z))	C_44_H_84_NO_8_P	↓	↓
28	-	Nbd-ceramide	C_30_H_49_N_5_O_6_	↑	↑
29	HMDB0112534	PS(20:0/22:4(7Z,10Z,13Z,16Z))	C_48_H_86_NO_10_P	↓	↓
30	HMDB0062769	Epsilon-caprolactam	C_6_H_11_NO	↓	↓
31	HMDB0061690	LysoPI(20:4(5Z,8Z,11Z,14Z)/0:0)	C_29_H_49_O_12_P	↑	↑
32	HMDB0243518	(-)-Norephedrine	C_9_H_13_NO	↑	↑
33	HMDB0256609	Piromidic acid	C_14_H_16_N_4_O_3_	↑	↑
34	-	Plaunotol	C_20_H_34_O_2_	↓	↓
35	-	2-Methyl-3-(pyrimidin-2-YL)propanoic acid	C_8_H_10_N_2_O_2_	↓	↓
36	HMDB0062789	1,1,3,3-tetramethylurea	C_5_H_12_N_2_O	↓	↓
37	HMDB0007970	PC (16:0/18:0)	C_42_H_84_NO_8_P	↓	↓
38	-	(3aR,4S,5R,6aS)-5-Hydroxy-4-(hydroxymethyl)hexahydro-2H-cyclopenta[b]furan-2-one	C_8_H_12_O_4_	↑	↑
39	-	Dioleoylphosphatidylcholine	C_44_H_84_NO_8_P	↓	↓
40	HMDB0256256	Pentaethylene glycol	C_10_H_22_O_6_	↑	↑
41	HMDB0113156	PE-NMe (18:1(9Z)/22:1(13Z))	C_46_H_88_NO_8_P	↓	↓
42	HMDB0036827	Sclareol	C_20_H_36_O_2_	↑	↑
43	-	Azelaoyl PAF	C_33_H_66_NO_9_P	↑	↑
44	HMDB0008037	PC(18:0/18:1(11Z))	C_44_H_86_NO_8_P	↓	↓
45	HMDB0113033	PE-NMe(15:0/22:2(13Z,16Z))	C_43_H_82_NO_8_P	↑	↑
46	HMDB0038959	2-Methyl-6-phytylhydroquinone	C_27_H_46_O_2_	↓	↓
47	HMDB0007988	PC(38:4)	C_46_H_84_NO_8_P	↓	↓
48	HMDB0041466	Melilotoside C	C_47_H_78_O_16_	↓	↓
49	HMDB0001060	Ubiquinol 8	C_49_H_76_O_4_	↓	↓
50	-	Nomifensine maleate	C_20_H_22_N_2_O_4_	↑	↑
51	HMDB0008046	PC(18:0/20:3(5Z,8Z,11Z))	C_46_H_86_NO_8_P	↓	↓
52	HMDB0011351	PE(P-16:0/20:3(8Z,11Z,14Z))	C_41_H_76_NO_7_P	↓	↓
53	-	Prostaglandin A1 ethyl ester	C_22_H_36_O_4_	↑	↑
54	HMDB0005779	1-(1Z-octadecenyl)-2-(5Z,8Z,11Z,14Z-eicosatetraenoyl)-sn-glycero-3-phosphoethanolamine	C_43_H_78_NO_7_P	↓	↓
55	HMDB0243889	1-Hexadecene	C_16_H_32_	↑	↑

Note: “↑” indicates an upward trend; “↓” indicates a downward trend. Abbreviations: DG, diacylglycerol; PI, phosphatidylinositol; PS, phosphatidylserine; Ser−Cys−Arg, serine–cysteine–arginine; LysoPC, lysophosphatidylcholine; PC, phosphatidylcholine; LysoPI, lysophosphatidylinositol; PE-NMe, monomethylphosphatidylethanolamine; PE, phosphatidylethanolamine.

**Table 3:** Differential metabolites related to reproductive toxicity identified in ESI^−^ mode.

No.	HMDB ID	Metabolite	Formula	Control vs. AEF	Model vs. AEF
1	HMDB0010644	PG(18:1(9Z)/22:6(4Z,7Z,10Z,13Z,16Z,19Z))	C_46_H_77_O_10_P	↓	↓
2	HMDB0113037	PE-NMe(15:0/24:0)	C_45_H_90_NO_8_P	↑	↑
3	-	8-Geranylumbelliferone	C_19_H_22_O_3_	↑	↑
4	-	13-Tetradecynoic acid	C_14_H_24_O_2_	↑	↑
5	-	LY-411575	C_26_H_23_F_2_N_3_O_4_	↑	↑
6	-	JWH-210 4-Hydroxypentyl (100 microg/mL in Methanol)	C_26_H_27_NO_2_	↑	↑
7	-	Tofacitinib	C_16_H_20_N_6_O	↑	↑
8	-	2′,5′-Dimethoxycinnamic acid	C_11_H_12_O_4_	↑	↑
9	-	(R)-4-((3S,5R,8R,9S,10S,13R,14S,17R)-3-hydroxy-4,4,10,13,14-pentamethyl-7,11-dioxohexadecahydro-1H-cyclopenta[a]phenanthren-17-yl)pentanoic acid	C_27_H_42_O_5_	↑	↑
10	HMDB0008431	PC(20:4(5Z,8Z,11Z,14Z)/18:0)	C_46_H_84_NO_8_P	↓	↓
11	-	Rapanone	C_19_H_30_O_4_	↑	↑
12	-	6,8a-Seco-6,8a-deoxy-5-oxoavermectin“1b” aglycone	C_33_H_46_O_7_	↑	↑
13	-	Aureothin	C_22_H_23_NO_6_	↑	↑
14	-	cis-EODA	C_18_H_34_O_3_	↑	↑
15	HMDB0039883	Ascorbyl palmitate	C_22_H_38_O_7_	↑	↑
16	HMDB0041446	Rosmaricine	C_20_H_27_NO_4_	↓	↓
17	-	Methyl farnesoate	C_16_H_26_O_2_	↑	↑
18	HMDB0002000	Myristoleic acid	C_14_H_26_O_2_	↑	↑
19	-	N-(Adamantan-1-yl)-1-(5-fluoropentyl)-1H-indole-3-carboxamide	C_24_H_31_FN_2_O	↑	↑
20	HMDB0000529	5Z-Dodecenoic acid	C_12_H_22_O_2_	↑	↑
21	-	2-C-methyl-D-erythritol 2,4-cyclodiphosphate	C_5_H_12_O_9_P_2_	↓	↓
22	-	5-Hydroxyconiferaldehyde	C_10_H_10_O_4_	↓	↓
23	HMDB0015263	Proguanil	C_11_H_16_ClN_5_	↓	↓
24	-	(+)-trans-Chrysanthemic acid	C_10_H_16_O_2_	↑	↑
25	-	Hydrocortisone acetate	C_23_H_32_O_6_	↓	↓
26	HMDB0004708	Pinellic acid	C_18_H_34_O_5_	↓	↓
27	HMDB0031031	2-Dodecylbenzenesulfonic acid	C_18_H_30_O_3_S	↓	↓
28	HMDB0035243	1-Phenyl-1,2-propanedione	C_9_H_8_O_2_	↓	↓
29	HMDB0035596	Armexifolin	C_15_H_18_O_4_	↑	↑
30	HMDB0013505	1-Stearoyl-2-vaccenoyl-sn-glycero-3-phospho-(1′-sn-glycerol-3′-phosphate)	C_42_H_82_O_13_P_2_	↓	↓
31	HMDB0002500	Bromide	Br^−^	↓	↓
32	HMDB0035828	Lactupicrin	C_23_H_22_O_7_	↓	↓
33	HMDB0000840	Salicyluric acid	C_9_H_9_NO_4_	↓	↓
34	-	3-(5,8-Dimethoxy-1,4-dioxonaphthalen-2-yl)sulfanylpropanoic acid	C_15_H_14_O_6_S	↑	↑

Note: “↑” indicates an upward trend; “↓” indicates a downward trend. Abbreviations: PG, phosphatidylglycerol.

## Data Availability

The original contributions presented in this study are included in the article. Further inquiries can be directed to the corresponding author(s).

## Abbreviations

The following abbreviations are used in this manuscript:

*T. wilfordii*	*Tripterygium wilfordii*
TG	Tripterygium glycosides
TGT	Tripterygium glycoside tablets
CKD	chronic kidney disease
AEF	Alkaloid-enriched fraction
NAEF	Non-alkaloid-enriched fraction
T	Testosterone
FSH	Follicle-stimulating hormone
LH	Luteinizing hormone
HPT	Hypothalamic–pituitary–testicular
PPI	Protein–protein interaction
GO	Gene Ontology
KEGG	Kyoto Encyclopedia of Genes and Genomes
BP	Biological processes
CC	Cellular components
MF	Molecular functions
PCA	Principal component analysis
OPLS-DA	Orthogonal partial least squares-discriminant analysis
FC	Fold change
DG	Diacylglycerol
PI	Phosphatidylinositol
PS	Phosphatidylserine
Ser-Cys-Arg	Serine–cysteine–arginine
LysoPI	Lysophosphatidylinositol
PE-NMe	Monomethylphosphatidylethanolamine
PE	Phosphatidylethanolamine
PG	Phosphatidylglycerol
ABP	Androgen-binding protein
GnRH	Gonadotropin-releasing hormone
HPG	Hypothalamic–pituitary–gonadal
17β-HSD	17β-hydroxysteroid dehydrogenase
LysoPC	Lysophosphatidylcholine
GPLs	Glycerophospholipids
PC	Phosphatidylcholine
PLA2	Phospholipase A2
AA	Arachidonic acid
PGs	Prostaglandins
COX	Cyclooxygenase
LDH	Lactate dehydrogenase
LOX	Lipoxygenase
PGE2	Prostaglandin E2
LTB4	Leukotriene B4
SPF	Specific-pathogen-free
CCK-8	Cell Counting Kit-8
ANOVA	One-way analysis of variance
